# Studying Grammar

**Published:** 1856-12

**Authors:** 


### STUDYING GRAMMAR.

JOSEPH T. BUCKINGHAM, one of the best of living writers and grammarians, once said that "Not one child in a thousand ever received the least benefit from studying the rules of grammar before he was fifteen years old."

We believe that countless thousands of dollars are more than thrown away, in defective modes of modern school-teaching. Children are put to studies long before their time—long before their minds are capable of comprehending their nature—and in the vain and painful effort to do it, disease is often engendered, by the premature and undue straining of the brain, to say nothing of that distaste and utter aversion to study, which is a very natural result, lasting sometimes for life, thus destroying, in embryo, minds which, had they been duly led, might have been the ornaments of any age.

We consider it a radical defect in our schools, that children are made to study branches which are above their comprehension, allied to an error not less mischievous, of being sent to school too early. A child should never be allowed to enter a school-room, not even a Sunday-school, if it has religious parents, until the seventh year, and for the next three years, should be allowed to study but one branch at a time, for a period of not over two hours at a time in the forenoon, and one in the afternoon; to have no studying to do at home, and be compelled to play in the open air, at least three hours after breakfast and two hours after dinner; the remainder of the time being expended in some pleasurable and useful handicraft.

From ten until sixteen, we would have them give four hours daily to brain work, learning one thing at a time, making thorough work of that one thing, so as never to have to learn it again, or unlearn a portion of it.

Be assured, it is for the want of some system like this, that so many of our children enter life with a general knowledge of many things, but knowing nothing critically or thoroughly; for after all, the only knowledge which makes us practically useful, is that which makes us acquainted with the minutiæ of any subject.

